# Cerebral hypoperfusion in post-COVID-19 cognitively impaired subjects revealed by arterial spin labeling MRI

**DOI:** 10.1038/s41598-023-32275-3

**Published:** 2023-04-10

**Authors:** Miloš Ajčević, Katerina Iscra, Giovanni Furlanis, Marco Michelutti, Aleksandar Miladinović, Alex Buoite Stella, Maja Ukmar, Maria Assunta Cova, Agostino Accardo, Paolo Manganotti

**Affiliations:** 1 Department of Engineering and Architecture, University of Trieste, Trieste, Italy; 2 Clinical Unit of Neurology, Department of Medicine, Surgery and Health Sciences, Trieste University Hospital-ASUGI, University of Trieste, Trieste, Italy; 3 Radiology Unit, Department of Medicine, Surgery and Health Sciences, Trieste University Hospital-ASUGI, University of Trieste, Trieste, Italy

**Keywords:** Biomedical engineering, Neurology

## Abstract

Cognitive impairment is one of the most prevalent symptoms of post Severe Acute Respiratory Syndrome COronaVirus 2 (SARS-CoV-2) state, which is known as Long COVID. Advanced neuroimaging techniques may contribute to a better understanding of the pathophysiological brain changes and the underlying mechanisms in post-COVID-19 subjects. We aimed at investigating regional cerebral perfusion alterations in post-COVID-19 subjects who reported a subjective cognitive impairment after a mild SARS-CoV-2 infection, using a non-invasive Arterial Spin Labeling (ASL) MRI technique and analysis. Using MRI-ASL image processing, we investigated the brain perfusion alterations in 24 patients (53.0 ± 14.5 years, 15F/9M) with persistent cognitive complaints in the post COVID-19 period. Voxelwise and region-of-interest analyses were performed to identify statistically significant differences in cerebral blood flow (CBF) maps between post-COVID-19 patients, and age and sex matched healthy controls (54.8 ± 9.1 years, 13F/9M). The results showed a significant hypoperfusion in a widespread cerebral network in the post-COVID-19 group, predominantly affecting the frontal cortex, as well as the parietal and temporal cortex, as identified by a non-parametric permutation testing (p < 0.05, FWE-corrected with TFCE). The hypoperfusion areas identified in the right hemisphere regions were more extensive. These findings support the hypothesis of a large network dysfunction in post-COVID subjects with cognitive complaints. The non-invasive nature of the ASL-MRI method may play an important role in the monitoring and prognosis of post-COVID-19 subjects.

## Introduction

There is growing evidence of a post COronaVIrus Disease 2019 (COVID-19) state, known as Long COVID, characterised by long-term complications or symptoms persisting for at least 4 weeks following Severe Acute Respiratory Syndrome COronaVirus 2 (SARS-CoV-2) infection, most recently defined as post-acute sequelae of SARS-CoV-2 or post-acute COVID-19^1–3^. Long term complications were observed in more than 30% of subjects affected by COVID-19^4^, including those who have had less severe forms of COVID-19 and the asymptomatics^5^. Post-COVID-19 results in a broad range of manifestations, affecting several organs and systems in the body, often including the central or the peripheral nervous system in a significant proportion of patients.

The most frequent neurological manifestations of post-COVID-19 include increased fatigue, diffuse myalgia, ageusia, anosmia, headache, sleep disturbances dysautonomia and cognitive impairment^1,6–9^. Particularly, cognitive impairment has being increasingly recognised as a long-term sequela of the COVID-19^7,10–13^. Recent follow-up investigations reported prevalence of cognitive deficits in 36% of patients at 3 months^10,11^ and 10 months^7^ after infection.

The mechanisms underlying the pathophysiology of post-COVID-19 neurologic symptoms are still debated. Advanced functional neuroimaging techniques may contribute to a better understanding of the pathophysiological brain changes in post-COVID-19 subjects by identifying the metabolic and perfusion alterations in affected brain regions. Several recent 18-fluorodeoxyglucose positron emission tomography (^18^FDG-PET) studies reported hypometabolism in several brain regions in post-COVID-19 patients with persistent functional symptoms, including cognitive deficit^12,14–19^. In the light of the detected metabolic alterations assessed with nuclear imaging techniques, the identification of brain perfusion patterns would provide additional information to better understand the underlying mechanisms of COVID-19 neurological sequelae. Cerebral blood flow (CBF) is correlated to cerebral metabolic rate and brain functional activity, since it affects the delivery of oxygen and nutrients to brain tissues^20^.

Arterial spin labeling (ASL) is a relatively new magnetic resonance imaging (MRI) technique to measure CBF, which is increasingly being used to investigate brain perfusion in subjects affected by neurological diseases^21–25^. This noninvasive MRI method uses arterial blood water as an endogenous tracer to measure tissue perfusion^26,27^ and it is able to detect affected brain regions in subjects with cognitive impairment^28^. Recently, an ASL study detected a hypoperfusion in subcortical regions in adults who previously self-isolated at home due to COVID-19 disease compared to non-COVID-19 controls who experienced flu-like symptoms^29^.

Thus, the advanced neuroimaging techniques and processing might contribute to identify the perfusion alterations underlying the symptoms of COVID-19 neurological sequelae.

The aim of the present study was to investigate regional cerebral perfusion abnormalities in post-COVID-19 subjects who reported a subjective cognitive impairment after a mild SARS-CoV-2 infection in comparison to healthy controls using an MRI-ASL technique.

## Materials and methods

### Study population and protocol

In this study, we investigated the brain perfusion alterations by MRI-ASL image processing in patients with neuro-cognitive sequelae of COVID-19. We included consecutive subjects admitted to the post-COVID neurological outpatient clinic of the University Hospital of Trieste, Italy, between 1 September 2021 and 31 January 2022 with presence of self-reported cognitive impairment in the post-acute COVID-19 period. The inclusion criteria was the persistent or ex-novo cognitive impairment at least after 4 weeks from Polymerase Chain Reaction (PCR) test-confirmed SARS-CoV2 infection. Exclusion criteria were: age < 18 or > 65 years, previous neurological or psychiatric diseases (i.e. major depressive disorder), neuroimaging assessed major vascular alterations, previous history of cognitive deficits, consumption of agents affecting the nervous system (e.g., antipsychotic, antidepressant or antiepileptic drugs). In addition, we excluded the patients who suffered from moderate-to-severe COVID-19 disease, defined as patients positive to SARS-CoV-2 with clinical and radiographic evidence of lower respiratory tract disease or hospitalised for COVID-19.

All subjects admitted to the post-COVID neurological outpatient clinic who reported a subjective cognitive impairment after the initial clinical evaluation, including medical history and comprehensive neurological examination, underwent Montreal Cognitive Assessment (MoCA) test for cognitive deficits and Fatigue Severity Scale (FSS).

All included patients underwent MRI imaging within 15 days from MoCA assessment. Imaging protocol included high resolution structural 3D T1-weighted images and 3D pseudocontinuous arterial spin labeling (3D-pcASL), as well as standard clinical brain imaging including diffusion weighted imaging (DWI) to screen the presence of acute ischemic lesions. ASL scans and maps were visually inspected by two experienced radiologists (M.U. and M.A.C) to exclude the presence of artifacts. A total of two subjects were excluded for large motion artifacts and 24 post COVID-19 subjects were included in the final analysis.

As many as twenty-two age- and sex-matched healthy controls, who underwent MRI imaging—including ASL—in our University Hospital during a previous project before the SARS-CoV-2 outbreak and had no history of cognitive impairment or of any other neurological disease, were retrospectively selected.

The research was conducted according to the principles of the Declaration of Helsinki. All participants released their informed consent for treatment of clinical data after all procedures had been fully explained, as for standard institutional procedure. This study was approved by the Local Ethics Committee CEUR (Comitato Etico Unico Regionale, FVG, Italy).

### Neuropsychological assessment

The MoCA was administered during the first visit by a trained neurologist using the validated Italian version^30^ and was further described by domain scores^31^ based on single item scores (orientation: spatial and temporal orientation; attention: digit span, letter A tapping, subtraction; executive: trail making, abstraction, word fluency; visuoconstructive: cube copying, clock drawing; language: naming, sentence repetition; memory: delayed word recall). The global MoCA test score was corrected for years of education (YoE; + 1 point if ≤ 12 YoE). Domain-scores were not adjusted for YoE. In addition, the MoCA score corrected according to correction for Italian population^32^ was also calculated.

The FSS, consisting of 9 sentences related to the interference of fatigue with daily activities and subjectively rating its severity on a 7-point scale (1 = “strongly disagree”; 7 = “strongly agree”), was administered during the visit^33^.

### MRI imaging and processing

All subjects were scanned on an Ingenia 3T MRI scanner (Philips Healthcare, Best, The Netherlands) using a 32-channel head coil. High-resolution whole brain anatomic images were acquired using 3D T1-weighted (3DT1w) scan with TR = 8.4 ms, TE = 3.9 ms, flip angle = 8°, voxel size = 1 × 1 × 1 mm^3^ and 180 slices.

ASL-MRI was performed using the pseudo-continuous arterial spin labeling (pcASL) with label duration = 1800 ms, post-labeling delay (PLD) = 2000 ms. 3D gradient and spin-echo (GraSE) pCASL scans with background suppression were obtained with TR/TE = 4.1 s/13 ms, FOV = 240 × 240 mm^2^, matrix size = 80 × 75, voxel size = 3 × 3 × 5 mm^3^, 20 slices, flip angle = 90°. Seven label/control image pairs were acquired as well as one calibration M0 image with no background suppression. All subjects were asked to rest and to keep their eyes closed during data acquisition. Post-COVID-19 and control subjects were acquired using exactly the same imaging protocol on the same MRI scanner.

The individual steps of ASL data processing and analysis were performed using FSL 6.0.5 (FMRIB, Oxford, United Kingdom) including Bayesian Inference for Arterial Spin Labelling MRI (BASIL)^34^. The head motion correction was carried out with MCFLIRT, non-brain tissue removal was performed with a brain extraction tool (BET). Spatial smoothing was also applied^35^. CBF maps were quantified from perfusion-weighted images (averaged pairwise subtracted control label images) by applying the general kinetic model^36^ according to the ASL white paper^26^ using voxel-wise calibration with the M0 image. To correct the partial volume effect spatially regularised technique was applied^37^. For each subject native space CBF maps were spatially normalised into the Montreal Neurological Institute—MNI152 standard-space (2-mm T1-weighted average structural template image).

### Data analysis

Voxelwise analysis was performed to identify statistically significant differences in CBF maps between post-COVID﻿-19 subjects and healthy controls by a non-parametric permutation testing^38^ (5000 permutations) using the FSL’s randomize tool. The resulting group difference maps were thresholded using a threshold-free cluster enhancement (TFCE) method^39^ and a family-wise error (FWE) corrected p-values < 0.05 for multiple comparisons across space.

In addition, information on regional perfusion values was extracted by means of a region of interest (ROI) analysis for nine selected cortical regions including frontal, parietal, temporal, and occipital lobes, as well as cerebellum. Anatomic cortical ROIs were defined by the Harvard–Oxford cortical structural atlas in MNI standard space. The mean values of CBF within the masked anatomic ROIs were calculated for each subject. The regional mean CBF values of the brain regions were compared between the two groups.

The differences between groups in age, sex, years of education, and neuropsychological MoCA Score were also tested.

Continuous variables with a normal distribution are presented as mean and standard deviations (mean ± SD), those with a skewed distribution as median and interquartile ranges (IQRs) indicating the first and third quartiles, and categorical variables as counts and percentages (%). Differences between groups were tested with Student’s t-test for normally distributed continuous variables, Mann–Whitney U-test for skewed variables, and Pearson’s Chi square for categorical variables. Level of significance was set at 0.05.

## Results

The demographic and clinical characteristics of the 24 post-COVID-19 patients with subjective cognitive impairment included in the study are reported in Table 1.

**Table 1:** Demographic and clinical characteristics of post-COVID-19 patents.

	(n = 24)
Age (years)	53.0 ± 14.5
Sex (M/F)	9M/15F
Education (years)	14.3 ± 3.2
Δ Covid-19 symptom—Post-Covid-19 assessment (days)	179.5 (128.5–210.5)
Δ Covid-19 symptom—MRI assessment (days)	10.8 ± 2.1
Pre-existing comorbidities and risk factors	
Ischemic heart disease	1 (4.2%)
Hypertension	6 (25.0%)
Atrial fibrillation	2 (8.3%)
Dyslipidemia	3 (12.5%)
Diabetes mellitus	2 (8.3%)
Obesity	4 (16.6%)
Smoke	3 (12.5%)
Autoimmune disease	1 (4.2%)
Clinical features during COVID-19 acute phase	
Fever	18 (75.0%)
Upper respiratory airways involvement	16 (66.6%)
Asthenia	13 (54.1%)
Myalgia/arthralgia	13 (54.1%)
Dyspnea	10 (41.7%)
Headache	9 (37.5%)
Hyposmia	9 (37.5%)
Hypo/dysgeusia	6 (25.0%)
Diarrhea/gastrointestinal distress	3 (12.5%)
Palpitations/tachycardia	2 (8.3%)
Post-COVID-19 manifestations	
Number of symptoms per patient	3.5 (2–5)
Asthenia	15 (62.5%)
Dyspnea	10 (41.7%)
Hyposmia	8 (33.3%)
Headache	6 (25.0%)
Myalgia/arthralgia	5 (20.8%)
Dizziness/gait instability	4 (16.7%)
Palpitations/tachycardia	4 (16.7%)
Hypo/dysgeusia	3 (12.5%)
Diarrhea/gastrointestinal distress	1 (4.2%)

There were no significant differences in age, sex and education between the post-COVID-19 (n = 24) and healthy control (n = 22) groups (53.0 ± 14.5 years for patients vs 54.8 ± 9.1 years for healthy subjects, p = 0.556; 62.5% of women for patients vs 59.1% of women for healthy subjects; p-value = 0.813; 14.3 ± 3.2 years of education for patients vs 14.9 ± 3.0 years of education for healthy subjects, p = 0.536).

Among the 24 included post-COVID-19 patients the reported pre-existing comorbidities and risk factors were hypertension (25.0%), obesity (16.6%), smoke (12.5%), dyslipidemia (12.5%), atrial fibrillation (8.3%), diabetes mellitus (8.3%), ischemic heart disease (4.2%) and autoimmune disease (4.2%). The prevalence of comorbidities and risk factors in the control group were hypertension (22.3%), obesity (13.6%), smoke (18.2%), dyslipidemia (13.6%), diabetes mellitus (4.5%), ischemic heart disease (4.5%) and autoimmune disease (9.0%).

During the acute phase of SARS-CoV-2 infection, main symptoms of included post-COVID-19 subjects were fever (75.0%), upper respiratory airways involvement (66.6%), asthenia (54.1%), myalgia/arthralgia (54.1%), dyspnea (41.7%), headache (37.5%), hyposmia (37.5%), hypo/dysgeusia (25.0%), diarrhea/gastrointestinal distress (12.5%), palpitations/tachycardia (8.3%). None of the included patients were hospitalised or underwent oxygen therapy during the acute phase.

Beside cognitive complaint, the post-COVID-19 manifestations reported during examination were asthenia (62.5%), persistent dyspnea (41.7%), hyposmia (33.3%), headache (25.0%), myalgia/arthralgia (20.8%), dizziness/gait instability (16.7%), palpitations/tachycardia (16.7%), hypo/dysgeusia (12.5%), diarrhea/gastrointestinal distress (4.2%).

All participants underwent standard clinical brain MRI assessment and none of them presented lesions in DWI or particular signs of atrophy or structural changes.

The median duration between post-COVID clinical evaluation, including the cognitive assessment, and the first infectious symptoms was 5.9 (4.2–6.9) months. The magnetic resonance imaging was performed within 15 days from cognitive assessment (10.8 ± 2.1 days).

The results of cognitive assessment are summarised in Table 2. The median MoCA was 26 (24–27). The median of MoCA score corrected according to correction for Italian population^32^ was 23.5 (22.4–24.9). None of the patients had a total corrected MoCA score below the cut-off for pathological impairment (< 18), according to the normative data for the Italian population^32^. However, corrected and uncorrected total MoCA scores was significantly lower in post-COVID-19 group compered to HC (p < 0.001). In particular, post-COVID-19 group presented significantly lower sub-domain MoCA scores for executive, attention, language and especially, memory functions, while no differences in orientation and visuoconstructive functions were observed (Table 2).

**Table 2:** Results of cognitive assessment of post-COVID-19 patents.

	post-COVID-19	Healthy controls	
	(n = 24)	(n = 22)	p-value
MoCA	26 (24–27)	29 (28–30)	< 0.001*
MoCA domain scores			
Orientation (max 6)	6 (6–6)	6 (6–6)	0.607
Attention (max 6)	5.5 (4.5–6)	6 (6–6)	0.006*
Language (max 6)	5 (5–6)	6 (5–6)	0.011*
Visuospatial function (max 4)	4 (4–4)	4 (4–4)	0.711
Memory (max 5)	3 (2–4)	5 (4–5)	< 0.001*
Executive function (max 4)	3.5 (3–4)	4 (4–4)	0.017*
MoCA corrected according Aiello et al.^32^	23.5 (22.4–24.9)	26.9 (26.3–27.2)	< 0.001*
Fatigue Severity Scale (FSS)	5.32 ± 1.26	–	–

*Statistically significant differences.

The average FSS score in post-COVID-19 group was 5.32 ± 1.26 and 45.8% presented a score higher than the FSS cut-off.

**Figure 1:**
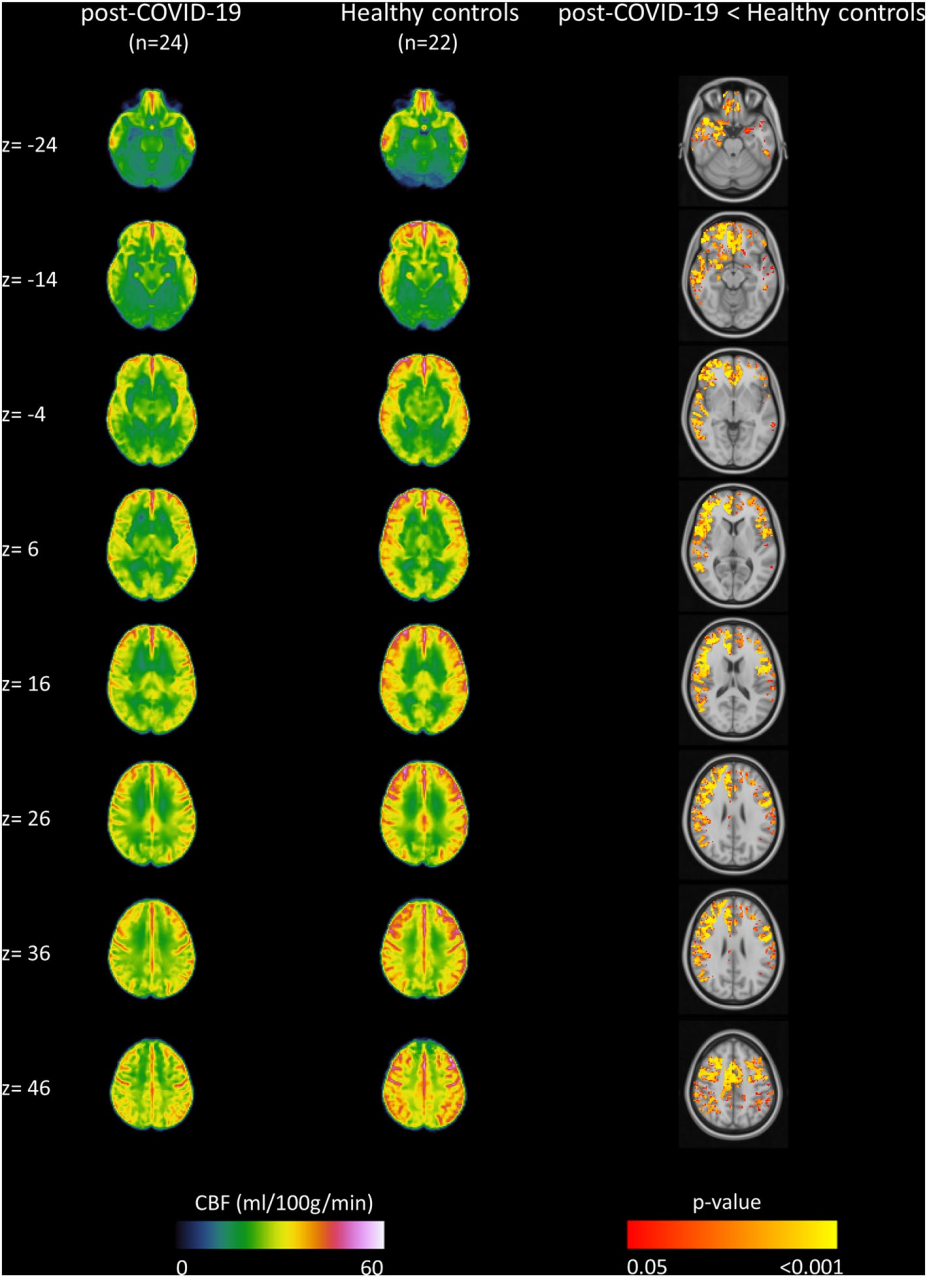
Result of group analysis of MRI-ASL data. The group averaged cerebral blood flow (CBF) maps (ml/100 g/min) calculated for the post-COVID-19 group (left column) and healthy subjects (middle column). Right column depicts regions that show significant hypoperfusion in post-COVID-19 patients compared to healthy controls (non-parametric permutation test, p < 0.05, FWE-corrected with TFCE). No regions with a significantly higher perfusion in post-COVID-19 patients compared to healthy controls were detected. Images are reported in the 2-mm MNI152 standard space and in radiological convention.

In Fig. 1 are shown the averaged CBF maps calculated for the post-COVID-19 group and healthy subjects, as well as the regions that showed a significant hypoperfusion in the post-COVID-19 group compared to the healthy controls as identified by a non-parametric permutation testing (p < 0.05, FWE-corrected with TFCE). The summary of cluster-level statistics for significant hypoperfused clusters is reported in Table 3. A lower CBF in the post-COVID-19 group compared to healthy subjects was detected in the right and left frontal, parietal and temporal cortex. The hypoperfusion areas identified in the right hemisphere regions were more extensive. No regions with a significantly higher perfusion in post-COVID-19 patients compared to healthy controls were detected.

**Table 3:** The summary of cluster-level statistics of the identified hypoperfused regions in post-COVID-19 patients compared to healthy controls (anatomical regions, cluster sizes, MNI coordinates of peak locations, as well as t-score and p-values).

Area	Side	Voxels	MNI coordinate			T score	p-value
			x	y	z		
Frontal lobe	R	13,296	12	40	− 8	6.69	0.001
Frontal lobe	L	7266	− 52	0	20	7.47	0.001
Parietal lobe	R	6525	58	− 34	22	7.59	0.001
Parietal lobe	L	1731	− 44	− 44	50	6.92	0.004
Temporal lobe	R	5206	62	− 22	− 8	6.02	0.001
Temporal lobe	L	978	− 52	− 34	− 20	7.23	0.002

The global mean of CBF in gray matter was lower in post-COVID group compared to controls (37.7 (33.3–44.8) vs 44.6 (40.8–48.7), p-value = 0.011). Table 4 reports the results of the ROI analysis of the selected anatomical regions. The mean CBF was significantly lower in left frontal, right frontal, right parietal, left temporal and right temporal lobes in post COVID-19 compared to healthy controls. No significant differences in regional mean CBF were observed for left parietal lobe, left and right occipital lobes, as well as for cerebellum. There was no significant correlation between the MoCA scores and CBF values within the post-COVID-19 group.

**Table 4:** Median (IQR) of regional mean CBF values (ml/100 g/min) of post-COVID-19 subjects and healthy controls at the various ROIs and comparison between two groups.

Area	Side	CBF		p-value
		Post-COVID-19	Healthy controls	
Frontal lobe	R	46.3 (40.6–55.3)	52.2 (49.5–61.9)	0.003*
Frontal lobe	L	47.7 (41.3–57.0)	53.4 (50.4–62.2)	0.021*
Parietal lobe	R	45.3 (39.4–53.4)	50.6 (49.3–58.7)	0.036*
Parietal lobe	L	47.1 (40.7–57.6)	52.6 (50.1–57.9)	0.078
Temporal lobe	R	34.7 (31.4–42.7)	41.7 (38.1–48.0)	0.005*
Temporal lobe	L	36.3 (30.8–45.6)	42.2 (38.8–50.7)	0.033*
Occipital lobe	R	36.9 (31.3–44.4)	38.0 (34.5–49.0)	0.220
Occipital lobe	L	39.0 (33.2–45.8)	41.0 (38.8–46.5)	0.392
Cerebellum	–	25.1 (21.3–34.4)	29.6 (26.5–35.2)	0.101

*Statistically significant differences.

## Discussion

The present voxel-based MRI-ASL study identified a significant hypoperfusion in frontal, temporal, and parietal cortex in post-COVID-19 subjects with persistent cognitive impairment 2–10 months after the initial symptoms. The identified hypoperfusion areas were more predominant in frontal regions and more extensive in the right hemisphere. These hypoperfusion clusters were highly discriminant to distinguish cognitively impaired post-COVID-19 patients and healthy subjects.

The identified hypoperfusion areas, in particular frontal lobes, could justify the primarily reduced cognitive performance in executive, attention, language and memory observed in post-COVID-19 patients. The cognitive complaints observed in our sample were in accordance with the common clinical picture reported in post-COVID-19 studies^7,14,40^. The same was observed in studies which encompassed non-hospitalised patients and those who did not suffer from severe COVID-19 infection^40,41^. In particular, deficits in memory^40–42^, attention^40–42^, language and executive functions^40,41^ domains have been observed in post-COVID-19 subjects. In general, such cognitive deficits were characterised by functional or structural impairment of the frontal and prefrontal lobes^43,44^. Indeed, frontal lobes represent key hubs for working memory, inhibition, cognitive flexibility, planning, and problem solving^43,44^.

These cognitive deficits have also been linked with hypometabolism pattern revealed by FDG-PET imaging in several studies performed on acute and post-COVID-19 patients that presented cognitive complaints^12,14,16,17^. Frontal and less extensive temporoparietal cortical hypometabolism was observed in seven acute COVID-19 patients with progressive normalization of cerebral metabolism during follow-up at 1 and 6 months, but with persisting prefrontal hypometabolism^17^. FDG-PET pattern characterised by prevailing frontal lobe hypometabolism, which may reflect an immune mechanism, was detected in four patients with COVID-19-related encephalopathy^16^. Predominant frontoparietal cortical hypometabolism was observed in 10 out of 15 patients, included subacute COVID-19 patients with decline in frontoparietal cognitive functions^14^. The follow-up FDG-PET performed on eight patients at 6 months after COVID-19 reveled a reduction of the initial frontoparietal and, to a lesser extent, temporal hypometabolism, although the alterations were still measurable^12^. The anatomical MRI performed in aforementioned studies has not detected specific abnormalities for most of the subjects^7,14,16,17^. However, the MRI study, which investigated longitudinal alterations before and after COVID-19, has detected significant effects, including a greater reduction in gray matter thickness and tissue contrast in the orbitofrontal cortex and parahippocampal gyrus, greater changes in markers of tissue damage in regions that are functionally connected to the primary olfactory cortex and a greater reduction in global brain size in the SARS-CoV-2 patients/subjects compared to controls^45^. The same study reported a greater cognitive decline between the two time points in the participants who were infected with SARS-CoV-2. A recent ASL study reported a significantly decreased CBF in the thalamus, orbitofrontal cortex and regions of the basal ganglia in subjects who previously self-isolated at home due to COVID-19 when compared against controls who experienced flu-like symptoms but tested negative for COVID-19^45^.

The inflammatory parainfectious process targeting specially the frontal lobes and/or frontal networks was suggested as the underlying cause of the reported clinical, neurophysiological and neuroimaging findings in COVID-19 patients^46^. Post-infectious inflammation, production of anti-neuronal autoantibodies, vasculitis, cytokine-related hyperinflammation and the cerebral complications of hypoxia and coagulopathy are possible underlying pathophysiological mechanisms induced by the SARS-CoV-2 infection^14,47^. Successful therapy with immunoglobulin was reported in two cases with status epilepticus in COVID-19 infection suggesting strong autoimmune mechanism^48^. The aforementioned pathophysiological mechanisms may be a valid explanation for cortical and blood brain barrier dysfunction, leading to cerebral hypopefusion, hypometabolism, and cognitive impairment in post-COVID-19 subjects. The novelty of this study is that we identified a hypoperfusion areas (frontal, temporal, and parietal lobes), similar to the previously reported FDG-PET hypometabolism pattern, in twenty-four post-COVID-19 subjects with persistent cognitive impairment by non-invasive ASL-MRI technique and analysis, without any radiological contrast agents and radiopharmaceuticals.

Our findings showed that ASL imaging and analysis were able to reveal cerebral hypoperfusion pattern in post-COVID-19 subjects with cognitive deficit. As a non-invasive technique, MRI-ASL could be a useful tool for the follow-up of such patients.

Our study is a single center study on a moderate study sample. The included patients were screened with MoCA test which do not provide comprehensive information about overall cognitive performance. ASL acquisition and subsequently the results of analysis can potentially be susceptible to the different labeling efficiency, which may vary over arteries, scans, and subjects. Variable time points from COVID-19 infection, i.e., range 2–10 months form COVID-19 symptoms onset, median (IQR) 6.0 (4.3–7.0 months), may pose another limitation. However, all patients reported cognitive complaints at the moment of the acquisition. The included patients had a paucisymptomatic acute COVID-19 which did not require hospitalisation or ventilatory support.

## Conclusions

In this study we identified a significative alteration of cerebral perfusion pattern in post-COVID-19 subjects who reported cognitive deficit by using a non-invasive ASL-MRI perfusion imaging technique and analysis. Particularly, significant hypoperfusion was observed predominantly in bilateral frontal, as well as in temporal, and parietal areas compared to healthy subjects, supporting the hypothesis of a large network dysfunction. The non-invasive nature of the ASL-MRI method may play an important role in the monitoring and prognosis of post-COVID-19 subjects.

## Data Availability

Anonymized data are available upon reasonable request to the corresponding author.
